# The Effect of Filial Piety and Cognitive Development on the Development of Adolescents' Depressive Symptoms: A Longitudinal Study

**DOI:** 10.3389/fpsyg.2021.751064

**Published:** 2021-10-27

**Authors:** Yingqiu Pan, Ruheng Tang

**Affiliations:** Institute of Psychology, School of Public Policy, Xiamen University, Xiamen, China

**Keywords:** reciprocal filial piety, authoritarian filial piety, cognitive autonomy, depression, academic pressure

## Abstract

The present study aims to investigate the pathways through which filial piety and cognitive development work on the development of depressive symptoms in adolescents as well as the trigger of adolescents' depressive symptoms (e.g., academic pressure). Two hundred fifty-seven Chinese adolescents (128 females and 129 males) participated in the study from Grade 7 to Grade 9. Results showed that both filial piety and cognitive autonomy significantly contribute to the development of adolescents' depressive symptoms and academic pressure. But reciprocal filial piety (RFP) and authoritarian filial piety (AFP) as two coexisting aspects of filial piety contribute to depressive symptoms in opposite directions. RFP provides significant protection against adolescents' depressive symptoms directly and indirectly through promoting the development of adolescents' cognitive autonomy and alleviating adolescents' academic pressure. In contrast, AFP positively contributes to adolescents' depressive symptoms by hindering the development of cognitive autonomy and intensifying academic pressure.

## Introduction

Adolescence is a critical transition period from childhood to adulthood. At this stage, adolescents must learn how to deal with psychosocial challenges in their daily interactions with surrounding environments, such as independence from parents and academic competition in school. Failure to manage various psychosocial challenges put adolescents at great risk for mental health problems, such as depression. Results of national mental health survey showed the prevalence of depression rises substantially throughout adolescence, with the cumulative probability of depression rising from 7.4% in early adolescence to as high as 25% by the end of adolescence (China National Mental Health Development Report, 2019–2020). Compared with normal adolescents, adolescents with depressive symptoms are more likely to develop psychiatric disorders or maladaptive behaviors in the long run, such as anxiety disorders, disruptive disciplinary behaviors, self-injurious and suicidal behaviors (Blakemore, 2019; Wang et al., 2021; Xu et al., 2021). Adolescents' vulnerability to depression and the negative consequence of depression for individual development call for a thorough understanding of the pathogenesis of adolescents' depressive symptoms as well as the process that may protect adolescents from depression.

Research showed that stress is an important trigger for depressive symptoms in adolescents (Fu et al., 2018). Among the various psychological stressors that may trigger depressive symptoms in adolescents, academic pressure merits extra attention. A recent meta-analysis study showed that academic pressure has a stronger association with Chinese adolescents' depressive symptoms as compared with loss-, health-, relationship-, and punishment-related stressful events (Miao, 2020). The strong bearing of academic pressure or low academic achievement on adolescents' depressive symptoms are also documented in various Eastern or Western nations, such as India, UK and Finland (Pelkonen et al., 2008; Jayanthi et al., 2015; López-López et al., 2021). It suggests that helping adolescents to better manage academic pressure is essential for protecting them from depression. In light of this, the present study aims to investigate the process of how individual and social factors protect adolescents from depression as well as academic pressure.

From an intrapersonal perspective, Biegler (2010) argued that whether an individual can successfully manage stressors largely depends on his/her capacity to reevaluate false negative thoughts associated with the stressful situation and employ “debiasing” or coping strategies to respond to stressors in an autonomous way. It implies that being able to autonomously reassess negative thoughts and feelings associated with academic learning in an adaptive manner is critical for adolescents to cope with academic pressure. Evidence from clinical studies also suggested that the efficacy of evidence-based psychotherapy and antidepressant medication for depression is closely related to their success in promoting autonomy in clients with depression (Biegler, 2008). In short, autonomy is an important enabling factor in protecting individuals against stressors as well as depressive symptoms. Given that adolescence is a period during which the drive for autonomy ramps up, it is sensible to assume that the rapid development of autonomy during adolescence may serve as an important protective factor against stressors such as academic pressure, and the development of depressive symptoms in adolescents.

From an interpersonal perspective, social relationship is another important predictor of individual mental health and well-being throughout lifespan (Feeney and Collins, 2015). Among the various social relationships, close parent-child relationships have been reported to be negatively linked to adolescents' academic pressure (Pan, 2015) and depressive symptoms (Rutten et al., 2016). Conversely, insecure attachment to primary caregivers plays a facilitative role in the development of depressive symptoms in children and adolescents (Spruit et al., 2020). Based on these findings, a supportive parent-child relationship can be an important factor in relieving adolescents' academic pressure and protecting them from depressive symptoms.

Although findings of previous studies suggested that personal autonomy and supportive parent-child relationships are protective against depressive symptoms in adolescents, however, the exact pathways through which autonomy and parent-child relationships work together to contribute to adolescents' depressive symptoms and academic pressure are unclear. To further shed light on the process, a 3-year longitudinal study is conducted to investigate how personal autonomy and the parent-child relationship characterized by filial piety work on the development of depressive symptoms in adolescents and its triggering factors, such as academic pressure.

### Autonomy and Depression

Although the importance of autonomy for individual growth is well-established, the conceptualizations of autonomy are far from consistent. Erikson (1963) defines autonomy as a child's learning to “act” for him/herself from a behavioral approach. Later researchers adopted a domain-specific view of behavioral autonomy and operationalized it as adolescents' decision-making on their own over conventional, prudential, personal or multifaced issues (Smetana et al., 2004). It was found that adolescents' perceived behavioral autonomy over personal and multifaced issues is a negative predictor of their depressive symptoms but such relationship is not found between behavioral autonomy in conventional/prudential domain and depressive symptoms (Smetana et al., 2004; Eagleton et al., 2016).

The emotional approach of autonomy conceptualizes autonomy as adolescents' emotional separation or detachment from their parents (Steinberg and Silverberg, 1986; Pace and Zappulla, 2010). It implies that social relatedness to significant others and autonomy development are incompatible. However, self-determination theory and a large body of empirical evidence suggested that social connectedness is an important facilitator of individual autonomy development (Ryan and Deci, 2000). Regarding the relationship between emotional autonomy and psychosocial development, previous findings are also inconsistent. Some authors reported a positive correlation while others identified a reverse one (Chou, 2000).

The perplexing findings of the associations between behavioral and emotional autonomy and depression counter to the well-established belief that the development of autonomy is important and healthy for adolescents' psychosocial development. Revisiting the traditional conceptualizations of autonomy and the relationship between autonomy and depression is necessary. Beckert (2007) proposed to conceptualize autonomy from a cognitive approach, defining cognitive autonomy as a person's capacity to evaluate, judge, and make decisions on events or information according to one's own volition. The findings of clinical research suggested that psychotherapy affording people with depression greater autonomy to reevaluate and manage negative thoughts related to stressful situations is important for them to learn to manage stressors and can better prevent the relapse of depressive symptoms in clients (Biegler, 2008, 2010). That is, improved cognitive autonomy may help people cope with stressors and depressive symptoms. So, the present study assumes that the development of cognitive autonomy during adolescence may provide adolescents protection against depressive symptoms directly and indirectly through helping them better cope with psychosocial stressors (e.g., academic pressure). The specific hypotheses are presented as follows:

H_1_: The development of cognitive autonomy has a direct negative prediction to the development of depressive symptoms in adolescents;
H_2_: The development of cognitive autonomy has an indirect negative prediction to the development of depressive symptoms in adolescents by helping them better cope with academic pressure.

### Filial Piety and Depression

In Chinese society, filial piety is the core tenet of ethics that guides the way Chinese children behave toward their parents and defines the characteristics of their relationship with parents. Yeh and Bedford (2003) proposed a dual model of filial piety which categorizes filial piety into two dimensions: reciprocal filial piety (RFP) and authoritarian filial piety (AFP). RFP emphasizes the warm and supportive interpersonal connections between parents and children. AFP emphasizes hierarchy and children's submission to parents. The model shifts the traditional investigation of filial piety from the focus on cultural norms of hierarchy to the inherent structure of the parent-child relationship, which provides an important medium to characterize the relationships between parents and children in cultures where the family is seen as an important basic unit of social relations (Bedford and Yeh, 2021).

Few studies examined the association between filial piety and adolescents' depression. However, previous research showed that adult children's (or caregivers') filial piety is a significant and negative predictor of old parents' depressive symptoms in different cultural groups, such as rural China (Yang and Wen, 2021) and the Greater Chicago area in the US (Li and Dong, 2018). For adolescents, it was found that RFP positively contributes to adolescents' psychosocial development, such as perceived life satisfaction and social competence, but AFP does not show a contribution (Leung et al., 2010). Based on these findings, it is hypothesized that RFP and AFP will contribute to adolescents' depression in opposite directions (see H_3_).

For school learning, research showed that RFP plays a significant facilitating role in college students' academic achievement and the endorsement of intelligence incremental beliefs (Chen and Wong, 2014), as well as study engagement and satisfaction (Rózycka-Tran et al., 2021) while AFP plays an inhibiting or trivial role. These findings suggested that the supportive and affective parent-child relationship embedded in RFP is important for students' academic development. In addition, it was found that supportive parent-child relationships can provide critical protection against elementary school students' academic pressure (Liao et al., 2021). Based on these findings, it is assumed that the warm and close parent-child relationships associated with RFP can help adolescents reduce academic pressure whereas hierarchy and submission associated with AFP will increase adolescents' academic pressure. Hypotheses (4) and (5) are proposed below.

H_3_: RFP and AFP have a positive and negative prediction to the development of depressive symptoms among adolescents, respectively.
H_4_: RFP negatively predicts the development of depressive symptoms in adolescents mediated through relieving adolescents' academic pressure;
H_5_: AFP positively predicts the development of depressive symptoms in adolescents mediated through increasing adolescents' academic pressure.

To develop a more thorough understanding of the process through which cognitive autonomy and filial piety regulated the development of depressive symptoms in adolescents, the association between filial piety and cognitive autonomy is also examined. Previous research suggested that parents' RFP beliefs significantly facilitate adolescents' autonomous motivation via parental autonomy granting whereas parents' AFP beliefs significantly inhibit adolescents' autonomous motivation via parental psychological control (Pan et al., 2013). In addition, enhanced autonomy is documented to be an important protective factor in the treatment of depression (Biegler, 2008). Based on these findings, the present study proposes two hypotheses regarding the association between filial piety and cognitive autonomy as follows:

H_6_: RFP negatively predicts the development of depressive symptoms in adolescents through fostering adolescents' cognitive autonomy;
H_7_: AFP positively predicts the development of depressive symptoms in adolescents through suppressing adolescents' cognitive autonomy.

## Method

### Participants

A total of 321 seventh grade students participated in the study. All participants were recruited from eight randomly selected classes in three middle schools in a city in southern China, with 2–3 classes from each school. Two hundred fifty-seven students (128 girls and 129 boys) participated in the study for three consecutive years. The sample attrition rate was between 8 and 10% in the second and third wave of data collection. The primary reasons for the sample attrition were that some students transferred to other schools and some were not available at the time of data collection. The mean age of participants in seventh grade was 13.26 years, SD = 0.55 years. Twenty-eight percentage of mothers and 32% of fathers had two or more years of higher education. To check whether the attrition sample was selective, the mean scores for the variables of interest were compared between the attrition and longitudinal samples. No significant mean differences were found.

### Procedure

Each wave of data collection was completed by the same experimenter during the Spring Semester of each school year. Students completed questionnaires in their classroom at the scheduled time. For each wave of data collection, all students were given 30 min to complete questionnaires. Participation was voluntary and each participant received a pen as a reward for his or her participation.

### Measures

Questionnaires or scales were used to assess participants' depressive symptoms, academic pressure, cognitive autonomy, RFP and AFP. Scales used to assess depression and cognitive autonomy were initially developed in English. Translated Chinese version of these scales have demonstrated good validity and reliability among Chinese adolescent samples in previous research. Participants completed all questionnaires or scales in Chinese.

#### Depression

Children's Depression Inventory (CDI, Kovacs and Beck, 1977) was used to assess adolescents' depressive symptoms and related feelings (27 items), such as sadness, pessimism, and sense of failure. CDI has been widely used in Chinese youth and adolescent samples (Hou et al., 2012). In the present study, the internal consistency reliability coefficients (Cronbach's α) for the CDI were 0.93, 0.92, and 0.92 in the 1st, 2nd, and 3rd waves of data, respectively.

#### Academic Pressure

The Questionnaire of Academic Pressure (Chen, 2004) was used to assess adolescents' perceived academic pressure, referring to the extent to which adolescents feel stressed in school-related activities including course work, academic competition from peers, and academic performance etc. (15 items), e.g., I often feel overwhelmed by academic tasks. The reliability coefficients (Cronbach's α) for the questionnaire were 0.85, 0.90, and 0.89 in the 1st, 2nd, and 3rd waves of data, respectively.

#### Cognitive Autonomy

Cognitive Autonomy Inventory (CAI, Beckert, 2007) was used to assess cognitive autonomy (20 items), representing adolescents' capacity to think independently, express opinions, make decisions, and evaluate thoughts. The inventory has demonstrated good validity and reliability among Chinese adolescent samples (Beckert et al., 2012). In the present study, the reliability coefficients (Cronbach's α) for the inventory were 0.85, 0.90, and 0.89 in the 1st, 2nd, and 3rd waves of data, respectively.

#### Reciprocal Filial Piety

The Respecting and Caring Parents Subscale of the Filial Piety Scale (Yeh and Yang, 2009) was used to assess RFP, referring to the extent to which adolescents show their respect and caring for parents' feelings and life, including 19 items, e.g., When I go out or get home, I report to my parents so that they won't worry. The reliability coefficients (Cronbach's α) for the subscale were 0.93, 0.93, and 0.95 in the 1st, 2nd, and 3rd waves of data, respectively.

#### Authoritarian Filial Piety

The Protecting and Upholding Honor for Parents Subscale of the Filial Piety Scale (Yeh and Yang, 2009) was used to assess AFP, representing the extent to which adolescents believe that they should avoid trouble and bring honor to their parents (8 items), e.g., “I rarely quarrel with siblings in front of my parents so as not to make them unhappy.” The reliability coefficients (Cronbach's α) for the subscale were 0.79, 0.77, and 0.81 in the 1st, 2nd, and 3rd waves of data, respectively.

## Results

Data analysis of the present study consists of two parts: (1) using a one -way repeated measures ANOVA to analyze the developmental trajectories of adolescents' depressive symptoms, academic pressure, RFP, AFP, and cognitive autonomy from Grade 7 to Grade 9; (2) using Hierarchical Linear Modeling (HLM; Raudenbush and Bryk, 2002) to analyze the associations between adolescents' depressive symptoms and its predictors.

### The Development of Adolescents' Depressive Symptoms and Its Predictors

As shown in Table 1, depressive symptoms in adolescents showed a declining trend from Grade 7 to Grade 9, *p* = 0.03, partial η^2^ = 0.01, with a significant decrease between Grade 8 and Grade 9, Bonferroni *t* = 2.74, *p* = 0.02. Cognitive autonomy showed a steady increase from Grade 7 to Grade 9, *p* < 0.001, partial η^2^ = 0.20. No significant changes were found in adolescents' academic pressure, reciprocal filial piety, and authoritarian filial piety. In addition, no gender differences were found in the developmental trajectories of depressive symptoms and its predictors.

**Table 1:** The development of adolescents' depressive symptoms and its predictors.

**Variables**	**Grade 7 *M (SD)***	**Grade 8 *M (SD)***	**Grade 9 *M (SD)***	***F***
Depressive symptoms	2.41 (0.66)	2.41 (0.67)	2.32 (0.64)	3.56^*^
Academic pressure	3.17 (0.68)	3.14 (0.79)	3.13 (0.74)	0.42
Reciprocal filial piety	3.93 (0.65)	3.87 (0.65)	3.92 (0.66)	1.78
Authoritarian filial piety	3.83 (0.66)	3.87 (0.63)	3.88 (0.58)	0.92
Cognitive autonomy	3.53 (0.56)	3.79 (0.53)	3.86 (0.53)	62.29^***^

* *p < 0.05*;

*** *p < 0.001*.

### The Prediction of Filial Piety and Cognitive Autonomy to the Development of Depressive Symptoms

HLM was used to analyze the prediction of filial piety and cognitive autonomy to the development of depressive symptoms in adolescents. First, the intraclass coefficient (ICC) was computed based on the null model. About 61% of the variance in depressive symptoms was accounted for by the inter-individual variables, ICC = τ_00_/($\tau_{00}+\sigma^{2}$) = 0.27/(0.27 + 0.17) = 0.61. The estimates of variance components for the null model were significant at 0.001 level, indicating that it is appropriate to use a two-level HLM model to analyze the longitudinal data.

To test the hypotheses about the mediating role of academic pressure in the relationship between filial piety and cognitive autonomy and the development of adolescents' depressive symptoms, three two-level HLM analyses were conducted in a row. In the first analysis, level-1 model examined the prediction of filial piety and cognitive autonomy to the development of depressive symptoms in adolescents at the intra-individual level, as shown in Equation (1). To justify the interpretation of B_0i_, the variable year in Equation (1) was centered (i.e., the number of years minus 1). When centered, B_0i_ represents the average level of depressive symptoms in adolescents at Grade 7. In the level-2 model, the dependent variables were the individual intercepts and slopes derived from the level-1 model (see Equation 2–6). Given that the results of previous one-way repeated measures ANOVA showed that there were no gender differences in the developmental trajectories of depressive symptoms in adolescents and its predictors, the inter-individual variable of gender was not included in level-2 model. As shown in Table 2, both RFP and cognitive autonomy had a significant negative contribution to the development of depressive symptoms in adolescents, *p*s < 0.001. No significant association between AFP and the development of depressive symptoms was found, *p* > 0.05.

**Table 2:** Results of HLM analyses for adolescents' depressive symptoms.

	**Model 1**		**Model 2**		**Model 3**	
	**Coefficient**	**S.E**.	**Coefficient**	**S.E**.	**Coefficient**	**S.E**.
**Fixed effect**						
Intercept, γ_00_	2.39^***^	0.02	3.15^***^	0.03	2.39^***^	0.02
Year, γ_10_	−0.06^***^	0.02	−0.04	0.02	−0.04^**^	0.02
Reciprocal filial piety, γ_20_	−0.35^***^	0.04	−0.17^*^	0.08	−0.30^***^	0.03
Authoritarian filial piety, γ_30_	0.03	0.04	0.20^**^	0.08	−0.09^**^	0.03
Cognitive autonomy, γ_40_	−0.40^***^	0.03	−0.44^***^	0.04	−0.26^***^	0.03
Academic pressure					0.32^***^	0.03
**Random effect**						
Intercept, u_0_	0.11		0.13		0.07	
Level 1, *r*_it_	0.14		0.24		0.12	

* *p < 0.05*;

** *p < 0.01*;

*** *p < 0.001*.

Level-1 model:


(1) $$\begin{array}{l} {\text{Y}}_{\text{it}}\left(\text{depression}\right)={\text{B}}_{0\text{i}}+{\text{B}}_{1\text{i}}\left(\text{Year}-1\right)\\ \;+{\text{B}}_{2\text{i}}\left(\text{reciprocal filial piety}\right)\\ \;+{\text{B}}_{3\text{i}}\left(\text{authoritarian filial piety}\right)\\ \;+{\text{B}}_{4\text{i}}\left(\text{cognitive autonomy}\right)+r_{\text{it}} \end{array}$$

Level-2 model:


(2) $$\begin{array}{l} {\text{B}}_{0\text{i}}=\gamma_{00}+\mu_{0} \end{array}$$

(3) $$\begin{array}{l} {\text{B}}_{1\text{i}}=\gamma_{10} \end{array}$$

(4) $$\begin{array}{l} {\text{B}}_{2\text{i}}=\gamma_{20} \end{array}$$

(5) $$\begin{array}{l} {\text{B}}_{3\text{i}}=\gamma_{30} \end{array}$$

(6) $$\begin{array}{l} {\text{B}}_{4\text{i}}=\gamma_{40} \end{array}$$

In the second analysis, level-1 model examined the prediction of filial piety and autonomy to adolescents' academic pressure, as shown in Equation (7). Level-2 model was consistent with Equation (2)–(6). Results showed that both RFP and cognitive autonomy had an inhibiting effect on adolescents' academic pressure while AFP showed a facilitating effect (see Model 2 in Table 2).


(7) $$\begin{array}{l} {\text{Y}}_{\text{it}}\left(\text{academicpressure}\right)={\text{B}}_{0\text{i}}+{\text{B}}_{1\text{i}}\left(\text{Year}-1\right)\\ \;+{\text{B}}_{2\text{i}}\left(\text{reciprocal filial piety}\right)\\ \;+{\text{B}}_{3\text{i}}\left(\text{authoritarian filial piety}\right)\\ \;+{\text{B}}_{4\text{i}}\left(\text{cognitive autonomy}\right)+r_{\text{it}} \end{array}$$

In the third analysis, level-1 model examined the prediction of filial piety, autonomy and academic pressure to the development of depressive symptoms in adolescents, as shown in Equation (8). Level-2 model was consistent with Equation (2)–(6). Results showed that RFP, AFP and cognitive autonomy all negatively contributed to the development of depressive symptoms. Adolescents' academic pressure has a positive contribution to depressive symptoms (see Model 3 in Table 2).


(8) $$\begin{array}{l} {\text{Y}}_{\text{it}}\left(\text{depression}\right)={\text{B}}_{0\text{i}}+{\text{B}}_{1\text{i}}\left(\text{Year}-1\right)\\ \;+{\text{B}}_{2\text{i}}\left(\text{reciprocal filial piety}\right)\\ \;+{\text{B}}_{3\text{i}}\left(\text{authoritarian filial piety}\right)\\ \;+{\text{B}}_{4\text{i}}\left(\text{cognitive autonomy}\right)\\ \;+{\text{B}}_{6\text{i}}\left(\text{academic pressure}\right)+r_{\text{it}} \end{array}$$

Putting the results of three HLM analyses together, it can be concluded that RFP and the development of cognitive autonomy provide strong protection against the development of depressive symptoms in adolescents directly and indirectly through alleviating adolescents' academic pressure. Notably, AFP was not associated with depressive symptoms when academic pressure was not taken into account. However, the relationship between AFP and depressive symptoms changed after considering academic pressure. That is, AFP demonstrated a negative contribution to the development of depressive symptoms in adolescents directly. Meanwhile, AFP positively contributed to depressive symptoms through intensifying adolescents' academic pressure. This suggests that academic pressure may play an important suppressing role in the relationship between AFP and depressive symptoms. That is, the direct inhibitory effect of AFP on depressive symptoms is counteracted by the indirect facilitative effect of AFP on depressive symptoms by intensifying adolescents' academic pressure.

### The Prediction of Filial Piety to Autonomy Development

To examine the correlations between filial piety and cognitive autonomy, a two-level HLM was conducted. ICC for the null model was calculated, τ_00_/(τ_00_ + σ^2^) = 0.31/(0.31 + 0.19) = 0.62. That is, 62% variance of cognitive autonomy was accounted for by inter-individual variables. The estimation of variance components for the null model was significant at 0.001 level. It suggested that a two-level HLM model is appropriate for the data analysis. The level-1 and level-2 models are presented below (see Equation 9–13). The results showed that RFP had a positive contribution to the development of cognitive autonomy in adolescents, γ_20_ = 0.15, *p* = 0.005, whereas AFP had an opposite contribution, γ_30_ = −0.14, *p* = 0.003.

Level-1 model:


(9) $$\begin{array}{l} {\text{Y}}_{\text{it}}\left(\text{cognitive autonomy}\right)={\text{B}}_{0\text{i}}+\beta_{1\text{i}}\left(\text{Year}-1\right)\\ \;+{\text{B}}_{2\text{i}}\left(\text{reciprocal filial piety}\right)\\ \;+{\text{B}}_{3\text{i}}\left(\text{authoritarian filial piety}\right) \end{array}$$

Level-2 model:


(10) $$\begin{array}{l} {\text{B}}_{0\text{i}}=\gamma_{00}+\mu_{0} \end{array}$$

(11) $$\begin{array}{l} {\text{B}}_{1\text{i}}=\gamma_{10} \end{array}$$

(12) $$\begin{array}{l} {\text{B}}_{2\text{i}}=\gamma_{20} \end{array}$$

(13) $$\begin{array}{l} {\text{B}}_{3\text{i}}=\gamma_{30} \end{array}$$

**Figure 1:**
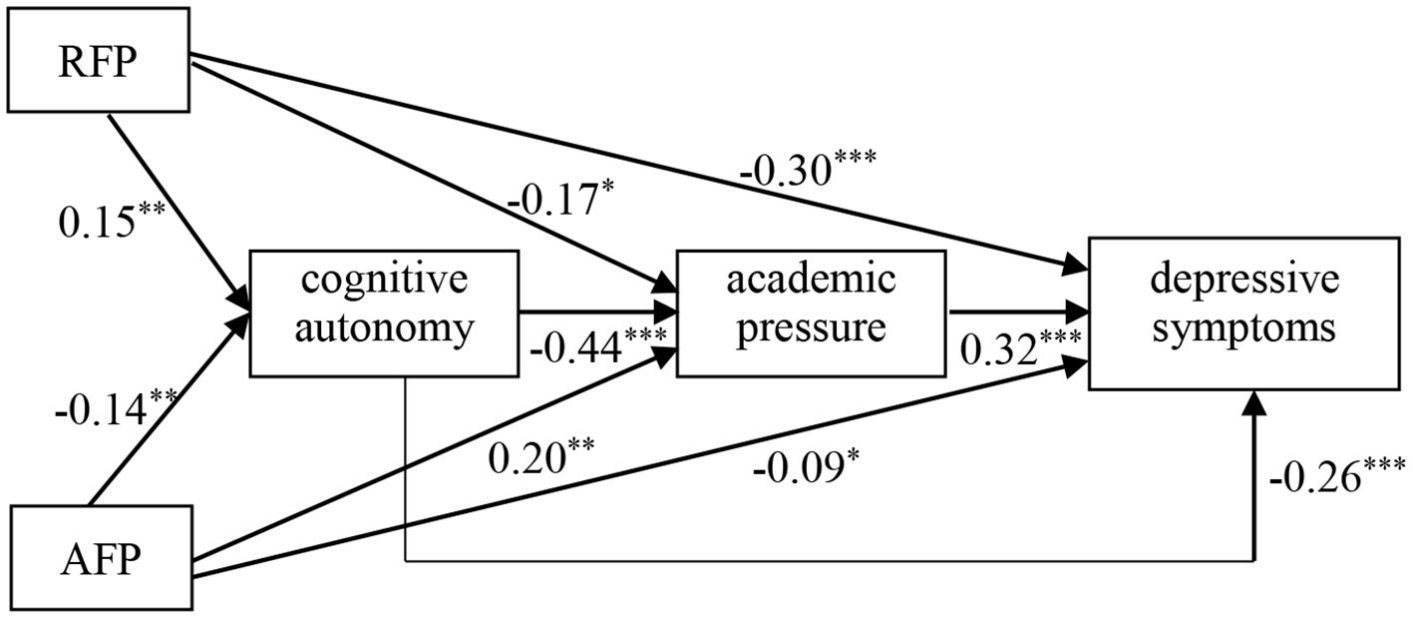
The prediction of filial piety and cognitive autonomy to adolescents' depressive symptoms. ^*^p < 0.05. ^**^p < 0.01. ^***^p < 0.001.

To better illustrate the associations between the development of depressive symptoms in adolescents and its predictors, the results of HLM analyses were summarized in Figure 1. Both filial piety and cognitive autonomy significantly contribute to the development of depressive symptoms in adolescents directly and indirectly through the mediating role of adolescents' academic pressure.

## Discussion

The present study investigated the pathways through which filial piety and cognitive autonomy shape the development of depressive symptoms in adolescents and their academic pressure during early adolescence. Consistent with the findings of previous studies (Miao, 2020), it was found that academic pressure in early adolescence is a significant positive contributor to the development of depressive symptoms in adolescents. Filial piety and cognitive autonomy contribute to the development of depressive symptoms in adolescents in multiple ways.

According to the dual filial piety model, caring and obedience are the core features of RFP and AFP, respectively. The results showed that the development of RFP and AFP was relatively stable from Grade 7 and Grade 9, indicating filial piety in terms of caring and obedience to parents is largely formed at a relatively young age. Notably, both RFP and AFP are associated with the development of depressive symptoms in adolescents. RFP is a reliable inhibitor, negatively contributing to depressive symptoms directly and indirectly through the mediated effect of academic pressure. Different from RFP, the relationship between AFP and depressive symptoms is somewhat complicated.

As shown in Table 2, when academic pressure was considered, the positive association between AFP and depression became negative. It suggested that academic pressure has a suppressing effect on the relationship between AFP and depression. That is, although AFP has a direct negative prediction to depression, AFP may increase adolescents' perceived academic pressure, which in turn, contributes to depression.

The finding of the associations of RFP and AFP with adolescents' depression extended the understanding of exiting literature on the relationship between filial piety and depression. That is, children's filial piety is not only important to their parents' depressive symptoms as disclosed in previous research (Yang and Wen, 2021) but also shapes the development of their own depressive symptoms. It is worthy to note that it is the emotional and caring component of filial piety or RFP that provides important protection against depressive symptoms rather than the obedient component of filial piety or AFP. The direct negative contribution of AFP on depression is largely counteracted by its indirect positive contribution to depressive symptoms through academic pressure.

Not surprisingly, cognitive autonomy showed a rapid increase in early adolescence. Results showed that the rapid growth of cognitive autonomy provides important protection against the development of depressive symptoms in adolescents during early adolescence. It not only inhibits depressive symptoms directly but also exerts an indirect inhibitory effect through attenuating adolescents' academic pressure. This suggests that cognitive autonomy is important for adolescents to evaluate academic stressful situations in a constructive way and cope with depressive feelings successfully. Additionally, RFP and AFP have a positive and negative contribution to the development of adolescents' cognitive autonomy, respectively. It indicates that children's emotional bonding and caring for parents is beneficial for the development of their cognitive autonomy whereas obedience to parents has an opposite effect.

One limitation associated with the present study is that it did not provide a broad examination of the association of emotional autonomy, behavioral autonomy, and cognitive autonomy with adolescents' depressive symptoms. A whole picture of how filial piety and autonomy contribute to the development of depressive symptoms in adolescents is still unclear. We expect that future studies may reconsider the traditional conceptualizations of emotional and behavioral autonomy to develop a more comprehensive understanding of parent-child relationships characterized by filial piety and individual growth in autonomy shape adolescents' depressive symptoms. In addition, the ICCs calculated based on the null HLM models suggested a high percentage of variance in adolescents' depressive symptoms is related to inter-individual variables but few inter-individual variables were included in the present study. More research efforts about the relationship between inter-individual variables and adolescents' depressive symptoms are expected in future studies.

Putting together, reciprocal filial piety and the growth of cognitive autonomy not only provide direct protection against the development of depressive symptoms in adolescents but also significantly attenuate the triggering effect of academic pressure on adolescents' depressive symptoms. In addition, RCP is also an important facilitator to the development of cognitive autonomy in adolescents. In contrast, AFP facilitates depressive symptoms through a negative contribution to the development of cognitive autonomy and a positive contribution to academic pressure. In conclusion, the cultural belief of filial piety adopted by Chinese adolescents is deeply intertwined with their psychosocial development and mental health. To promote adolescents' healthy development and well-being, more parenting emphasis should be placed on children's tending and caring for their parents or RCP and less emphasis on children's obedience to parents in academic learning.

## Data Availability Statement

The original contributions presented in the study are included in the article/supplementary material, further inquiries can be directed to the corresponding author/s.

## Ethics Statement

The studies involving human participants were reviewed and approved by Xiamen University. Written informed consent to participate in this study was provided by the participants' legal guardian/next of kin.

## Author Contributions

YP: responsible for data collection, data analysis, and manuscript writing. RT: responsible for format checking and proofreading. All authors contributed to the article and approved the submitted version.

## Funding

This study was supported by the Fundamental Research Funds for the Central Universities (2072021076).

## Conflict of Interest

The authors declare that the research was conducted in the absence of any commercial or financial relationships that could be construed as a potential conflict of interest.

## Publisher's Note

All claims expressed in this article are solely those of the authors and do not necessarily represent those of their affiliated organizations, or those of the publisher, the editors and the reviewers. Any product that may be evaluated in this article, or claim that may be made by its manufacturer, is not guaranteed or endorsed by the publisher.
